# The impact of ‘grounds’ on abortion-related outcomes: a synthesis of legal and health evidence

**DOI:** 10.1186/s12889-022-13247-0

**Published:** 2022-05-10

**Authors:** Fiona de Londras, Amanda Cleeve, Maria I. Rodriguez, Antonella F. Lavelanet

**Affiliations:** 1 Birmingham Law School, University of Birmingham, Birmingham, B15 2TT UK; 2 Women’s and Children’s Health, Karolinska Institute, Stockholm, Sweden; 3 Development and Research Training in Human Reproduction (HRP), Department of Sexual and Reproductive Health and Research, UNDP-UNFPA-UNICEF-WHO-World Bank Special Programme of Research, World Health Organization, Geneva, Switzerland; 4 Department of Obstetrics and Gynecology, Oregon Health and Science University, Portland, Oregon USA

**Keywords:** Abortion, Human rights, Abortion: grounds, Abortion: exceptional grounds, Abortion regulation, Abortion law, Abortion on request

## Abstract

Where abortion is legal, it is often regulated through a grounds-based approach. A grounds-based approach to abortion provision occurs when law and policy provide that lawful abortion may be provided only where a person who wishes to have an abortion satisfies stipulated ‘grounds’, sometimes described as ‘exceptions’ or ‘exceptional grounds’. Grounds-based approaches to abortion are, prima facie, restrictive as they limit access to abortion based on factors extraneous to the preferences of the pregnant person. International human rights law specifies that abortion must be available (and not ‘merely’ lawful) where the life or health of the pregnant woman or girl is at risk, or where carrying a pregnancy to term would cause her substantial pain or suffering, including but not limited to situations where the pregnancy is the result of rape or incest or the pregnancy is not viable. However, international human rights law does not specify a grounds-based approach as the way to give effect to this requirement. The aim of this review is to address knowledge gaps related to the health and non-health outcomes plausibly related to the effects of a grounds-based approach to abortion regulation. The evidence from this review shows that grounds have negative implications for access to quality abortion and for the human rights of pregnant people. Further, it shows that grounds-based approaches are insufficient to meet states’ human rights obligations. The evidence presented in this review thus suggests that enabling access to abortion on request would be more rights-enhancing than grounds-based approaches to abortion regulation.

**Supplementary Information:**

The online version contains supplementary material available at 10.1186/s12889-022-13247-0.

## Background

Where abortion is at least partially legal, it is commonly regulated through a grounds-based approach [1]. A grounds-based approach occurs when law and policy provides that lawful abortion may be availed of only where a person who wishes to have an abortion satisfies stipulated ‘grounds’, sometimes described as ‘exceptions’ or ‘exceptional grounds’. Grounds are defined as ‘circumstances under which abortion is lawful, that is, allowed or not contrary to law, or explicitly permitted or specified by law’ [2].

Typical ‘grounds’ include risk to the life or health of the pregnant woman, if the pregnancy results from rape, incest or sexual violence, severe or fatal fetal anomaly, or socio-economic grounds. However, grounds can be expressed in legal texts in ‘vague and confusing’ ways, making them even more difficult to implement [2]. In many cases these grounds-based approaches exist alongside and interact with other regulatory interventions such as gestational limits, third party authorization requirements, and criminalization to form a complex law and policy framework for abortion provision.

Grounds based approaches to abortion are, prima facie, restrictive as they limit access to abortion based on factors extraneous to the preferences of the pregnant woman. International human rights law (IHRL) specifies that abortion must be available (and not ‘merely’ lawful) where the life and health of the pregnant woman or girl is at risk, or where carrying a pregnancy to term would cause her substantial pain or suffering, including but not limited to situations where the pregnancy is the result of rape or incest or the pregnancy is not viable ([3], para 4). However, even though IHRL says abortion must be available in these circumstancs this does not mean that grounds-based approaches to abortion regulation are either mandated by or sufficient to satisfy IHRL. Rather, in ensuring that abortion is available in at least these circumstances states must be cognizant of, and must comply with, their broader IHRL obligations. These include obligations to take steps to ensure women do not have to undergo unsafe abortion ([4], para 6), to reduce maternal morbidity and mortality [3, 5], to effectively protect women and girls from the physical and mental risks associated with unsafe abortion [3, 5], to ensure that women’s and girls’ right to privacy and confidentiality in sexual and reproductive healthcare is protected, and to respect, protect and fulfil the broader right to the highest attainable standard of physical and mental health, including sexual and reproductive health.

The aim of this review is to address knowledge gaps related to the health and non-health outcomes that, while not directly linked through simple linear causal pathways, are plausibly related to the effects of a grounds-based approach to abortion regulation. The review followed a methodology for integrating human rights in guideline development that has been described elsewhere [6]. This methodology is well-suited to interventions that are complex, may have multiple interacting components, may be non-linear in their effects, and are often context dependent [7]. Such complex interventions often interact with one another, such that outcomes related to one individual or community may be dependent on others, and may be impacted positively or negatively by the people, institutions and resources that are arranged together within the larger system in which they are implemented [8]. This review was conducted as part of the evidence base for the World Health Organization (WHO)‘s recently-published *Abortion Care Guideline* [9]. It is one of seven reviews of evidence undertaken by the research team following the same methodological approach.

Throughout this review, and consistent with the approach in the *Abortion Care Guideline* ([9], p. xxii), we use the terms women, girls, pregnant women [and girls], pregnant people, and people interchangeably to include all those with the capacity to become pregnant.

## Methods

### Identification of studies and data extraction

This review examined the impact of the intervention of grounds-based approaches on two populations (i) people seeking abortion, and (ii) health professionals. Our study outcomes and search strategy were developed together with experts working in the fields of reproductive health, law, policy, and human rights. Our outcomes of interest included both health and non-health outcomes that, based on a preliminary assessment of the literature [10], could be linked to the effects of the ‘grounds’ intervention. Outcomes linked to those seeking abortion included delayed abortion, continuation of pregnancy, opportunity costs, reproductive coercion, and disproportionate impact. Outcomes linked to medical professionals included stigmatization, workload implications, referral of patients, and system costs.

We searched the databases PubMed, HeinOnline, and JStor and the search engine Google Scholar. The WHO’s *Safe Abortion: technical and policy guidance for health systems* (2nd edition) [11] included data up until 2012, thus, we limited our search to papers published in English after 2010 to the end of November 2020. We did not restrict on study design. We included only manuscripts that undertook original data collection or analysis i.e., quantitative studies (comparative and non-comparative), qualitative and mixed-methods studies, reports, PhD theses, and economic or legal analyses.

The full review team was comprised of four members (AC, FdL, MR and AL). FdL and AL developed the PICO question (People/Population/Patient; Intervention; Comparison (if applicable); Outcomes of Interest). Two reviewers (AC and FdL) conducted an initial screening of the literature. Titles and abstracts were first screened for eligibility using the Covidence® tool; full texts were then reviewed. A third reviewer (AL) confirmed that these studies met inclusion criteria. Two reviewers (FdL and AC) extracted data. Any discrepancies were reviewed and discussed with two additional reviewers (AL and MR). The review team resolved any discrepancies through consensus.

In order to fully understand the implications of the findings for abortion law and policy, we applied human rights standards to the data extracted from these manuscripts. The applicable standards were drawn from a review of the corpus of IHRL in accordance with the methodological approach applied [6]. They thus exclude regional and national human rights laws. The applicable standards were considered together with the evidence from the included manuscripts in order to identify, (a) which international human rights standards are engaged by grounds, (b) whether the evidence suggests that grounds have positive or negative effects on the enjoyment of rights, and (c) where no data are identified from the manuscripts against outcomes of interest, whether IHRL provides evidence that can further elucidate the impacts and effects of grounds. This is summarized in Tables 2 and 3 below.

### Analysis

Data from included studies were matched to the outcomes and presented in Evidence tables. In these tables the impact of each finding on the outcome was presented, as well as an overall conclusion of the identified findings across outcomes of interest. To summarize the effect of the intervention, across all study designs, we utilized a previously developed approach and incorporated a visual representation of effect direction. The direction of the evidence was illustrated by a symbol which indicated whether, in relation to that particular outcome, the evidence extracted from a study suggested an increase (▲), decrease (⊽), or no change in the outcome (○). The symbol did not indicate the magnitude of the effect [6, 8].

## Results

**Fig. 1:**
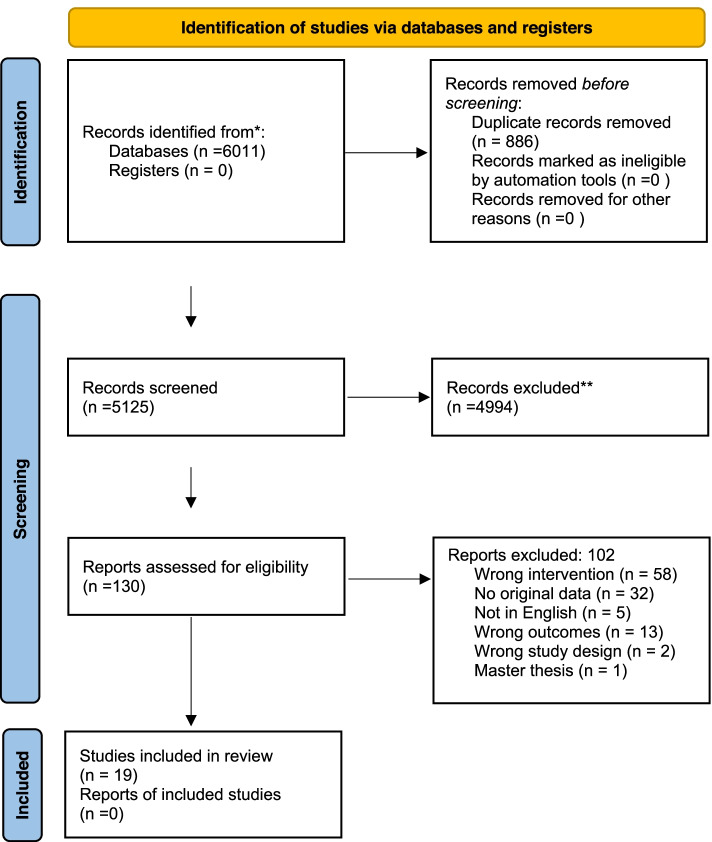
Prisma Flow diagram [12]

The initial search generated 5123 citations after duplicates were removed. We screened the titles and abstracts and conducted a full text screening of 130 manuscripts. After applying our exclusion criteria, 19 manuscripts were included in the final analysis (Fig. 1. Prisma flow diagram).

Manuscripts described data from the following settings: Argentina [13], Australia [14–18], Chile [19, 20], Colombia [21–23], Ethiopia [24], Ghana [25], Ireland [26, 27], Mexico [23, 28], Rwanda [29, 30], Thailand [31], United Kingdom/Great Britain [23, 32], and Uruguay [33]. The characteristics of included manuscripts are presented in Table 1. For the impact on people seeking abortion we included studies containing information relevant for the outcomes: delayed abortion [14, 20, 21, 27, 30] continuation of pregnancy [28, 33], opportunity costs [13–16, 20–24, 26, 29–32], self-managed abortion [26, 32], unlawful abortion [25, 26, 32], reproductive coercion [18, 24] and disproportionate impact [16, 18, 19, 23, 31]. For the impact on health professionals we included studies containing information relevant to workload implications [13, 18, 19, 24, 25], referral to another provider [14], imposition on conscience or ethics [19, 24], stigmatization [17], and system costs [18, 19, 27, 28, 33]. No evidence was identified linking the intervention to the outcomes: family disharmony, exposure to violence or exploitation, or impact on the provider-patient relationship.

**Table 1:** Characteristics of included studies

Author/year	Country	Methods	Participants
Aiken 2019 [32]	UK, Northern Ireland	Qualitative individual in-depth interviews (*n* = 30).	Women in Northern Ireland who had sought an abortion by travelling to a clinic in Great Britain or by using online telemedicine to self-manage an abortion at home.
Aiken 2018 [26]	Ireland	Qualitative individual in-depth interviews (*n* = 38).	Women in Ireland who had sought an abortion by travelling abroad to a clinic or by self-managing an abortion at home.
Aitken 2017 [27]	Ireland	Cross sectional study (*n* = 184).	Non-consultant hospital doctors training in Obstetrics and Gynaecology.
Amado 2010 [21]	Colombia	Case series (*n* = 36)	Women seeking legal advice after being denied a legal abortion or being subjected to unjustified care delays.
Antón 2018 [33]	Uruguay	Times series design (*n* = 93,762 births).	Data from the Perinatal Information System on planned and unplanned births.
Arnott 2017 [31]	Thailand	Mixed methods: legal analysis, cross sectional survey (*n* = 32) and individual interviews (*n* = 6). Informal interviews (*n* = not reported).	Key informants (government workers, healthcare providers, advocates from the non-profit sector (individual interviews) and members of a safe abortion programme (survey).
Black 2015 [14]	New South Wales and Queensland, Australia	Qualitative individual in-depth interviews (*n* = 22).	Physicians involved in abortion provision in the two states working in maternal-foetal medicine, sexual health, obstetrics and gynaecology, and family planning.
Casas 2017 [19]	Chile	Legal analysis and qualitative individual interviews (*n* = 61);	Hotline providers, healthcare providers, women with experiences of “illegal abortions”, their friends, partners and relatives
Clarke 2016 [28]	Mexico	Times series design. Analysis of vital statistics data covering live births (*n* = 23,151,080) and maternal deaths (*n* = 11,858) among women aged 15–44).	N/A.
DePiñeres 2017 [22]	Colombia	Qualitative individual in-depth interviews (*n* = 21) .	Women 16–24 years old who were denied an abortion due to their gestational age.
Diniz 2014 [16]	Brazil	Cross sectional survey (*n* = 1690) and in-depth interviews (*n* = 50) with participants who responded to the survey.	Obstetrician-gynaecologists aged 25–84 years affiliated with the Brazilian Federation of Obstetrics and Gynaecology. In-depth interviews were conducted with physicians who had provided abortions for women and girls who had been raped.
Küng 2018 [23]	Great Britain, Colombia, and Mexico.	Mixed methods: descriptive review of publicly available records and individual in-depth interviews (*n* = 17).	Interviews with key informants which included healthcare providers, academic scholars and representatives of non-governmental organizations.
LaRoche 2021 [15]	Australia	Qualitative individual in-depth interviews (*n* = 22).	Interviews with women, transgender and gender non-binary persons aged 19–46.
Madeiro 2016 [17]	Brazil	Mixed methods: cross sectional survey (*n* = 68) and individual in-depth interviews (*n* = 82).	Survey among 68 institutions providing legal abortion services and interviews with health care professionals (nurses, nurse technicians, physicians, social workers, psychologists).
Maira 2019 [20]	Chile	Mixed methods; qualitative individual in-depth interviews (*n* = 62), focus group discussions (*n* = 7) and a survey (*n* = 136).	Healthcare professionals (physicians, midwives, psychologists and social workers), healthcare union representatives and women.
McLean 2019 [24]	Ethiopia	Individual in-depth interviews (*n* = 31) and focus group discussions (*n* = 3).	Healthcare providers, 23–42 years old, involved in any aspect of abortion care including nurses, midwives, physicians, health officers, medical students and a pharmacist.
Mirlesse 2013 [18]	Brazil	Ethnographic observations (*n* = 80) and interviews (*n* = 9).	Observations of consultations in ultrasound scan and foetal medicine, prenatal genetics, prenatal paediatrics, and interviews with physicians.
Påfs 2020 [29]	Kigali, Rwanda	Qualitative individual interviews (*n* = 32) and focus group discussions (*n* = 5)	Healthcare providers (physicians, nurses and midwives) involved in post abortion care at three public hospitals.
Payne 2013 [25]	Ghana	Qualitative individual in-depth interviews (*n* = 4) and focus group discussion (*n* = 1).	Physicians providing abortion care.
Ramos 2014 [13]	Argentina	Mixed methods: Cross sectional survey (*n* = 157) and individual interviews (*n* = 27).	Healthcare providers (“physicians and non-physicians”) providing care within obstetrics and gynaecology.
Sahin Hodoglugil 2017 [30]	Rwanda	Mixed methods: review of hospital records (retrospective *n* = 2644, prospective *n* = 311), legal records (*n* = not reported), individual in-depth interviews (*n* = 22) and focus group discussions (*n* = 3).	Women aged 18–45 years, key informants including healthcare providers, representatives of courts, Ministry of Health, Ministry of Justice, and civil society organizations.

### Impact of grounds on abortion seekers

A summary of the impacts of grounds-based approaches on abortion seekers and the application to human rights are presented in Table 2. Evidence identified per study and outcome are presented in Supplementary Tables 1 and 2.

**Table 2:** Overall conclusions on the impact of grounds on abortion seekers

Outcome	Overall conclusion of evidence (A)	Application of HR standards (B)	Conclusion evidence + HR (C)
Delayed abortion	Overall, the findings from 6 studies indicate that grounds-based laws may contribute to abortion delays in different ways due to inconsistencies in interpretation and implementation of the legal grounds. Abortion delays can occur when: abortion medications are seized by customs; the process of obtaining a legal abortion through local ethics committees or courts is protracted; women’s rape claims are questioned; healthcare providers misapply the right to conscientious objection; there is disagreement among healthcare providers about severity of foetal anomaly; medical professionals wait until the health condition is severe enough that the woman’s condition is deemed life threatening.	Grounds-based laws engage states’ obligation to respect, protect and fulfil the rights to life and health (by taking steps to reduce maternal mortality and morbidity including by addressing unsafe abortion, by protecting people seeking abortion, and by ensuring abortion regulation is evidence-based and proportionate).	Grounds-based laws can result in delayed access to abortion care, including waiting until health conditions deteriorate to satisfy a ‘ground’. Such delays may be associated with unsafe abortion or increased risks of maternal mortality or morbidity. Where such delays increase risks of maternal mortality or morbidity, they have negative implications for rights.
Continuation of pregnancy	Overall, the findings from 2 studies indicate that grounds-based laws may indirectly contribute to continuation of pregnancy and thus increased fertility. When grounds-based laws are removed, and 1st trimester abortion is allowed on request, these studies demonstrated a decrease in fertility, possibly due to a reduction in unplanned births.	Grounds-based laws engage states’ obligation to respect, protect and fulfil the rights to life and health (by taking steps to reduce maternal mortality and morbidity including by addressing unsafe abortion, by ensuring that where it is lawful abortion is safe and accessible), and the right to decide the number and spacing of children. Grounds-based laws can also be a violation of the state’s obligation to ensure abortion is available where the life and health of the pregnant person is at risk, or where carrying a pregnancy to term would cause her substantial pain or suffering, including where the pregnancy is the result of rape or incest or where the pregnancy is not viable.	Grounds-based laws may result in continuation of pregnancy and unwanted birth. Grounds that have a disproportionately negative effect on the health and physical and mental integrity of abortion seekers, including on a woman’s ability to decide whether or not to continue with pregnancy, have negative implications for rights. Failure to ensure grounds do not result in denial of therapeutic abortion has negative implications for rights.
Opportunity costs	Overall, the findings from 15 studies, suggest that grounds-based laws may contribute to opportunity costs in several ways including: the need to travel for an abortion, increased financial costs, emotional stress and trauma, fear of/experienced judgement and stigma, bureaucratic and costly protracted legal processes, increased morbidity, being subjected to “interrogations” and having one’s rape claim questioned, unsafe abortions, having to carry an unwanted pregnancy or a pregnancy with severe malformations, to term. The findings from some of these studies point to an inconsistency in how grounds are interpreted and applied, which sometimes leads to unpredictability and inequity in terms of abortion access and healthcare quality for the abortion seeker. The findings from other studies indicate that certain grounds, such as health and rape grounds, are consistently interpreted very restrictively, which ultimately leads to the denial of an abortion.	Grounds-based laws engage states’ obligation to respect, protect and fulfil the rights to life and health (by ensuring that where it is lawful abortion is safe and accessible, by protecting people seeking abortion, and by ensuring abortion regulation is evidence-based and proportionate).	Grounds-based laws may operate in a way that imposes significant opportunity costs on people seeking abortion, and in a way that makes lawful abortion inaccessible in practice.
Unlawful abortion	Overall, evidence from 3 studies suggest that grounds-based laws may contribute to unlawful abortion.	Grounds-based laws engage states’ obligation to respect, protect and fulfil the rights to life and health (by taking steps to reduce maternal mortality and morbidity including by addressing unsafe abortion, and by protecting people seeking abortion).	Grounds-based laws may be associated with recourse to unlawful abortion. Where such unlawful abortions increase risks of maternal mortality or morbidity, grounds have negative implications for rights.
SMA	Overall, evidence from 2 studies suggest that grounds-based laws may contribute to self-managed abortion.	Grounds-based laws engage states’ obligation to respect, protect and fulfil the rights to life and health (by taking steps to reduce maternal mortality and morbidity including by addressing unsafe abortion, and by protecting people seeking abortion).	Grounds-based laws may be associated with recourse to unlawful abortion, including unlawful self-managed abortion. Where such unlawful abortions increase risks of maternal mortality or morbidity grounds have negative implications for rights.
Reproductive coercion	Overall, the findings from 2 studies suggest that grounds-based laws may contribute to reproductive coercion through the denial of an abortion.	Grounds-based laws engage states’ obligation to respect, protect and fulfil the rights to life and health (by ensuring abortion regulation is evidence-based and proportionate, by ensuring that provider refusal does not hinder access to abortion, and by ensuring that where it is lawful abortion is safe and accessible), the right to decide the number and spacing of children. Grounds-based laws can also violate of the state’s obligation to ensure abortion is available where the life and health of the pregnant person is at risk, or where carrying a pregnancy to term would cause her substantial pain or suffering, including where the pregnancy is the result of rape or incest or where the pregnancy is not viable.	Grounds-based laws that contribute to reproductive coercion through the denial of lawful abortion (as a result of unnecessary procedures or non-rights compliant interpretation and application), denial of therapeutic abortion, and denial of abortion in case of rape or incest have negative implications for rights.
Disproportionate impact	Overall, the findings from 5 studies suggest that grounds and grounds-based laws may have a disproportionate, negative impact on women with fewer resources, rural women and women with lower education, as well as those seeking abortion due to rape and on health grounds.	Grounds-based laws engage states’ obligation to respect, protect and fulfil the right to equality and non-discrimination. Grounds-based laws can also violate of the state’s obligation to ensure abortion is available where the life and health of the pregnant person is at risk, or where carrying a pregnancy to term would cause her substantial pain or suffering, including where the pregnancy is the result of rape or incest or where the pregnancy is not viable.	Grounds-based laws impact disproportionally on certain groups of women, including women who seek abortion following rape or therapeutic indication. This disproportionate impact has negative implications for the right to equality and non-discrimination in the provision of sexual and reproductive healthcare.
Family disharmony	No evidence identified	N/A	N/A
Exposure to violence or exploitation	No evidence identified	Grounds-based law may engage states’ obligation to respect, protect and fulfil the rights to privacy, health, and life.	Grounds-based approaches to the provision of abortion may require the disclosure of personal information to persons or institutions without clinical justification. In some cases, disclosure of such information may expose abortion seekers to risks of interpersonal violence, ostracisation or other harms (e.g., where a claim must be disclosed in order to access abortion) with negative implications for her right to privacy, health, and potentially right to life.

The evidence from six studies [14, 20, 21, 26, 27, 30] suggests that grounds-based approaches contribute to delays in accessing abortion. This includes delays resulting from inconsistencies in interpretation and application of grounds [21], questioning or ‘verification’ of women’s claims relating to grounds [14, 21], disagreement between medical professionals about whether a ground is satisfied [20], and health professionals delaying abortion until the pregnant person’s health condition is severe enough that the woman’s condition is deemed life threatening (and thus that a ‘risk to life’ ground is fulfilled) [20, 25]. The human rights standards reviewed make it clear that states must take steps to reduce maternal mortality and morbidity ([3, 5], paras [8, 10, 24, 30-33]), and to ensure that lawful abortion is safe and accessible [3]. The evidence from the reviewed studies suggests that grounds are associated with delay in a way that indicates incompatibility with these human rights obligations.

Evidence from two studies suggests that grounds-based laws may indirectly contribute to continuation of pregnancy [28, 33]. When grounds-based laws are removed, and first trimester abortion is allowed on request, these studies demonstrated a decrease in fertility, possibly due to a reduction in unplanned births [28, 33]. If, as these studies suggest might be the case, grounds are associated with unwanted continuation of pregnancy, they have clear implications for the right to decide on the number and spacing of children, as well as the right to privacy and to the highest attainable standard of physical and mental health. This is reinforced by the findings from two studies, which suggest that grounds-based laws may contribute to reproductive coercion through the denial of an abortion [18, 24]. This includes people who seek abortion in circumstances where IHRL requires it to be available, namely where the life and health of the pregnant woman or girl is at risk, or where carrying a pregnancy to term would cause her substantial pain or suffering [3]. Denial of abortion in such circumstances has been found to amount to torture, cruel, unusual, and degrading treatment or punishment, including where abortion on the grounds of fatal fetal impairment was unlawful [34, 35].

States have a long-standing obligation in IHRL to ensure that their regulatory choices—in this case grounds-based approaches to abortion—do not force women to resort to unsafe abortion and, if necessary, to review, reform and liberalize laws to ensure this ([3, 36], para 28). Three studies indicate that grounds-based approaches are associated with people availing of unlawful abortion per se [25–27], while two indicate an association with self-managed abortion [26, 32]. Self-managed abortion is not necessarily ‘unsafe’, but when availed of outside of the formal legal system and without the availability of appropriate and high-qualify information, medicines (including for pain management), and, where it is desired, support from trained health workers within the health system it can be understood as *less safe* [37]. Thus, grounds-based approaches engage states’ obligation to review, reform and liberalize their laws to reduce recourse to unsafe abortion ([36], para 28).

Evidence from 15 studies shows that grounds-based approaches contribute to opportunity costs [13–17, 20–24, 26, 29–32]. These costs are varied and include the need to travel for an abortion [26, 27], increased financial costs, emotional stress and trauma [26, 27] fear or experience of judgement and stigma [15], bureaucratic and costly protracted legal processes [14, 21, 29, 30], increased morbidity, being subjected to ‘nterrogations’ and having one’s rape claim questioned [16], unsafe abortions, and having to carry an unwanted pregnancy or a pregnancy with severe malformations to term [18].

The findings from some of these studies point to an inconsistency in how grounds are interpreted and applied, which sometimes leads to unpredictability and inequity in terms of abortion access and healthcare quality for the abortion seeker [13, 20, 23, 31] and requirements to provide legally-unnecessary documentation or ‘proof’ of fulfilling a ground [13, 17, 19]. One study shows that women who could obtain legal support and advice considered it vital to their ability to navigate the system of abortion provision [22]. In some cases, women who sought abortion based on a recognized ground reported verbal abuse and denial of services [21], and women who had to travel to access abortion sometimes reported reluctance to seek post-abortion care in cases of complications later [32]. The findings from other studies indicate that certain grounds, such as health and rape grounds, are consistently interpreted very restrictively, leading to the denial of abortion [16, 17, 21–24, 29, 30]. These studies indicate that a grounds-based approach is not sufficient to ensure that the state’s obligation to ensure abortion is lawful and available when continuing with pregnancy would cause substantial pain and suffering is fulfilled ([3], para 4).

five studies suggests that grounds-based approaches have a disproportionate impact on some groups of women [16, 18, 19, 23, 31] and thus undermine the right to equality and non-discrimination which is fundamental to the right to the highest attainable standard of physical and mental health. These studies suggest that in a grounds-based system of abortion law those with fewer resources [19], rural women, and women with lower education [18], as well as those seeking abortion due to rape [16] and on health grounds [23] are relatively less able to access abortion than other women.

### Impact of grounds on health professionals

A summary of the findings of grounds-based approaches on health professionals and the application of human rights are presented in Table 3. Evidence identified per study and outcome are presented in Supplementary Tables 1 and 2.

**Table 3:** Overall conclusions on the impact of grounds on health professionals

Outcome	Overall conclusion of evidence (A)	Application of HR standards (B)	Conclusion evidence + HR (C)
Workload implications	Overall, the findings from 5 studies suggest that grounds and grounds-based laws may have workload implications including: difficulties in interpreting and applying the law, preparing detailed files for court reviews, stress and fear of legal repercussions, and a frustration with the system when a diagnosis of a non-lethal foetal malformation can be made but abortion is not permitted.	Grounds-based laws engage states’ obligation to respect, protect and fulfil the rights to life and health (by ensuring abortion regulation is evidence-based and proportionate, and by protecting healthcare professionals providing abortion care).	Workload implications arising from grounds-based laws significant burdens on healthcare professionals providing abortion care, with negative implications for both their rights and the rights of persons seeking to access comprehensive abortion care.
Referral to another provider	Overall findings from 1 study suggest that grounds-based laws may contribute to referrals to another provider; physicians must make referrals to providers in another state to circumvent existing obstacles including ethics committees and other protracted processes.	Grounds-based laws engage states’ obligation to respect, protect and fulfil the rights to life and health (by protecting people seeking abortion).	Referrals to a provider in another jurisdiction may mitigate difficulties of access produced by grounds-based laws for those with resources and capacity to undertake travel.
Imposition on conscience or ethics	Overall, the findings from 2 studies indicate that grounds and grounds-based laws may contribute to providers experiencing an imposition on their conscience or ethics in two ways, either by a) resulting in the questioning of whether or not a provider should provide a legal abortion, or b) by preventing providers from giving women diagnosed with a foetal malformations an option to end their pregnancy.	Grounds-based laws engage states’ obligation to respect, protect and fulfil the rights to life and health (by protecting healthcare professionals providing abortion care).	Grounds-based laws may result in providers being required to deny abortion where provision would align with their conscience or ethics, or to declare a ground to have been satisfied in order to ensure safe abortion provision even where it may not strictly satisfy the requirements of the law. In both cases, there are negative implications for the provider.
Stigmatia sation	Overall, the findings from 1 study indicate that grounds-based laws may contribute to stigmatisation of healthcare providers who ultimately choose not to involve themselves in abortion care for this reason.	Grounds-based laws engage states’ obligation to respect, protect and fulfil the rights to life and health (by protecting healthcare professionals providing abortion care).	Decisions about whether to provide abortion care can have stigmatising and career limiting effects where grounds-based laws differentiate between the lawfulness of ‘reasons’ for accessing abortion, with negative implications for both providers’ rights and the rights of persons seeking to access abortion.
System costs	Overall, the findings from 5 studies suggest that grounds and grounds-based laws may contribute to system costs by indirectly contributing to continuation of pregnancy and maternal mortality, and directly by imposing costs on court systems, increased workloads of healthcare professionals, and by delaying care for pregnant women with severe health conditions.	Grounds-based laws engage states’ obligation to respect, protect and fulfil the rights to life and health (by taking steps to reduce maternal mortality and morbidity including by addressing unsafe abortion, by ensuring abortion regulation is evidence-based and proportionate, and by protecting people seeking abortion).	Grounds-based laws are associated with poor health outcomes and system costs and thus with exposure of abortion seekers to substantial costs and risks, with negative implications for rights.
Impact on provider-patient relationship	No studies identified	N/A	N/A

Evidence from five studies suggests that grounds have workload implications for health professionals [13, 18, 19, 24, 25] including difficulties in interpreting and applying the law [24], preparing detailed files for court reviews [18], stress and fear of legal repercussions [25], and frustration with the system when a diagnosis of a non-lethal fetal malformation has been made but abortion is not permitted [18, 19]. Findings from one study suggest that, in order to circumvent obstacles posed by or emanating from grounds-based approaches, referrals may be made to another provider operating in a setting where the ground in question does not preclude access to abortion [14]. One study suggests that where grounds do not permit abortion in situations of diagnosed fetal impairment, health professionals experience this as an imposition on their conscience or ethics [19]. Indeed, in another study the evidence showed that providers perceive grounds-based approaches as an imposition on their conscience or ethics to the extent that they question whether or not they should provide a legal abortion [24]. Findings from one study indicate that grounds-based laws may contribute to stigmatization of abortion provision and healthcare professionals ultimately choosing not to involve themselves in abortion care for this reason [17]. Evidence from five studies suggests that grounds-based laws may contribute to system costs. Indirect contributions to system costs are indicated by continuation of pregnancy and maternal mortality [19, 29, 33]. Direct contributions to system costs include the imposition of costs on court systems, increased workloads forhealthcare professionals [18], and delays to care for pregnant women with severe health conditions [27].

Evidence of the impact of grounds on health professionals points not only to their conditions of work but also to the implications of grounds for the health system and, thus, for the right to the highest attainable standard of physical and mental health. That right obliges states to ensure that health-care facilities, goods, and services are available, accessible, acceptable and of good quality ([38], paras 4, 12).

Overall, the evidence from these studies suggests that grounds-based approaches to abortion regulation undermine the right to health by impacting on health workers and the health system so as to make sexual and reproductive healthcare *less* available, accessible, and acceptable and to lower its quality.

## Discussion

Making abortion available based on grounds, sometimes after a limited period during which it is available without restriction as to reason, is a common approach to law and policy on abortion. To illustrate, as of February 2019 there were only 16 jurisdictions that prohibited abortion in all circumstances, and seven more where laws prohibited unlawful abortion but failed to specify expressly the grounds for lawful abortion [1]. States that have recently transitioned from near-total bans on abortion to some or wide legal availability have tended to adopt a grounds-based approach [39–41]. In addition, several constitutional and other courts have recently intervened to determine the constitutionality of abortion law in specified circumstances [42–49]. It is clear that using grounds as a mode of designing abortion provision is persistent. However, the evidence presented in this review suggests that this approach is suboptimal from a rights-based perspective. While modes of providing abortion care may vary as a pregnancy develops, whether and if so how abortion is provided should not be determined by legally prescribed grounds but by the preferences of the pregnant person and evidence-based clinical and service delivery protocols [9].

The evidence from this review shows that grounds-based approaches implicate states’ obligation to ensure that abortion is lawfully available where carrying a pregnancy to term would cause a pregnant woman substantial pain or suffering, where there is a risk to life or health, where pregnancy is a result of rape or incest, or where the pregnancy is not viable ([3], para 4). This is because grounds-based approaches to abortion provision are per se restrictive and are commonly subject to narrow interpretation, limitation and burdensome procedures so that ‘qualification’ for abortion under such grounds is very difficult to establish. As this can lead to women having recourse to less safe or unsafe abortions, grounds-based approaches engage states’ obligation to take steps, including revising their laws, to ensure women do not have to undergo unsafe abortion ([3, 4], para 6), to reduce maternal morbidity and mortality, and to effectively protect women and girls from the physical and mental risks associated with unsafe abortions. The evidence in this review points towards allowing abortion on request as the most effective approach to ensuring that abortion is available in the circumstances required by IHRL. In addition to being an effective mode of satisfying pregnant people’s rights, this would also mean that women are not required—and do not feel obliged—to provide reasons or justifications for their decision to end their pregnancies. Instead of defining only certain circumstances where abortion is permitted and thus, implicitly, justifiable, and the person seeking it deserving of abortion care, the provision of abortion without restriction as to reason would center the pregnant person’s reproductive autonomy and support her in giving effect to her assessment of what is right for her, regardless of circumstances. This is consistent with established frameworks for quality of care as well as with IHRL. Indeed, the WHO has now recommended against grounds-based approaches and recommended that abortion be made available on the request of the pregnant person [9].

## Limitations

This review has limitations. While the studies relate to 13 settings, this is nevertheless limited when compared to the number of jurisdictions that take a grounds-based approach to abortion [1]. This review also only contains manuscripts published in English. Further research on the impact of grounds in a wider range of settings would be valuable.

The realization of human rights applicable to abortion-related interventions is not a research area that readily lends itself to randomized controlled trials or comparative observational studies; rather, studies are often conducted without comparisons. While this may be considered a limitation from a standard methodological perspective for systematic reviews, it does not limit the ability to identify the human rights law implications of grounds-based approaches. Thus, while standard tools for assessing risk of bias or quality, including GRADE [50], were unsuitable, given the objective of fully integrating human rights implications into our understanding of the effects of grounds as a regulatory intervention, it was appropriate to engage with a wide variety of sources. Finally, and consistent with the methodological approach pursued [6], this review applies international, rather than regional or domestic, human rights law to develop a general understanding of the rights-related implications of grounds. The applicability of any individual human rights standard in a specific setting will depend on factors including the state’s ratification of relevant human rights instruments and their status in domestic law ([9], p. 7).

## Conclusions

The evidence from this review shows that grounds-based approaches to abortion limit access to abortion both because they exclude some women due to non-satisfaction of grounds *and* because they can operate in a way that makes availability and accessibility narrower in practice due to the chilling effects of continued criminalization, the burdens on providers, and the space they leave for conservative and limiting interpretation. Combining the evidence from the review of studies with evidence of applicable human rights standards indicates that grounds-based approaches result in violations of human rights. This points to the regulatory and human rights value of ensuring access to abortion on request of the pregnant person as a rights compliant, autonomy-based, and effective step in putting in place a supportive law and policy framework to support an enabling environment for quality abortion care.

## Supplementary Information

**Additional file 1: Suppl Table 1.** Evidence table: Impact on the intervention on abortion seekers. **Supple Table 2.** Evidence Table: The impact of the intervention on health professionals.

## Data Availability

All data generated or analysed during this study are included in this published article and its supplementary information files.

## Abbreviations

IHRL: International Human Rights Law
WHO: World Health Organization
