# Supplementary material for: The impact of ‘grounds’ on abortion-related outcomes: a synthesis of legal and health evidence
Source: BMC Public Health. 2022 May 10;22:936. doi: 10.1186/s12889-022-13247-0 (PMC9092771; doi:10.1186/s12889-022-13247-0)
Supplement: Supplementary file 1 — Additional file 1: Suppl Table 1. Evidence table: Impact on the intervention on abortion seekers. Supple Table 2. Evidence Table: The impact of the intervention on health professionals. [file 12889_2022_13247_MOESM1_ESM.docx]

*Suppl Table 1. Evidence table: Impact on the intervention on abortion seekers*

| **OUTCOME: DELAYED ABORTION** | | | |  |
| --- | --- | --- | --- | --- |
| **Studies** | **Direction of the evidence** | **What does this mean?** | **Overall conclusion** |  |
| Aiken 2019^1^ | ▲ | Grounds-based laws (preserve life and prevent grave and permanent damage to physical or mental health) may contribute to delayed abortion. Out of 30 women interviewed, 18 self-managed their abortions by ordering abortion medications online. Some women had their packages seized in customs and for others the deliveries were delayed by several weeks. One woman experienced long delays while waiting to be deemed legally eligible. | Overall, the findings from 6 studies indicate that grounds-based laws may contribute to abortion delays in different ways due to inconsistencies in interpretation and implementation of the legal grounds. Abortion delays can occur when: abortion medications are seized by customs; the process of obtaining a legal abortion through local ethics committees or courts is protracted; women’s rape claims are questioned; healthcare providers misapply the right to conscientious objection; there is disagreement among healthcare providers about severity of foetal anomaly; medical professionals wait until the health condition is severe enough that the woman’s condition is deemed life threatening. |  |
| Aitken 2017^2^ | ▲ | Grounds-based laws (real and substantial risk to life only) may contribute to delayed abortion; 33 physicians reported having been involved in the management of women who had an abortion because of severe or life-threatening illness. Of those, 27% reported that they had delayed the abortion until a pregnancy was deemed a "real and substantial risk" to life. |  |  |
| Amado 2010^2^ | ▲ | Grounds-based laws (life, rape, incest, health, fatal foetal malformations, “unwanted insemination”) may contribute to abortion delays due to inconsistencies in the interpretation and implementation of the health indication, when a woman’s rape claims are questioned or the right to conscientious objection is misapplied. In a review of 46 cases, 36 women were denied an abortion or obtained an abortion only after a protracted bureaucratic process; of those, 18 (50%) sought abortion on rape grounds and 22 experienced delays. The average delay from date of first request to abortion = 16 days (range 2 to 44) and in cases of rape = 79 days (range 37 to 150). |  |  |
| Black 2015^1^ | ▲ | In settings where grounds-based laws apply (threat to life*), and where local ethics committees are assigned the responsibility to determine legal eligibility for pregnancies >20 weeks or “in complex cases”, the case review process women go through is sometimes lengthy and contributes to abortion delays.  *Defined through case law as including threats to physical and mental health, including changes to future social and/or economic circumstances that might affect health. |  |  |
| Maira 2019^3^ | ▲ | Where grounds-based laws apply (life, rape, fatal foetal anomaly), access to an abortion may depend on the understanding of the law and its application by healthcare professionals. Participants reported that there are sometimes disagreements within a medical team about whether a foetal anomaly is incompatible with life or whether there is a risk to life, despite both being grounds for access. In these cases, abortion is denied or delayed until the health condition is severe enough that health professionals can agree that the condition is life threatening. |  |  |
| Sahin Hodoglugil 2017^3^ | ▲ | Where grounds-based laws apply (life, rape, forced marriage, incest); one healthcare provider reported a case where a minor sought an abortion on rape grounds but where she ended up giving birth before the legal process was concluded. |  |  |
| **OUTCOME: CONTINUATION OF PREGNANCY** | | | |  |
| Antón 2018 | ▲ | Shifting from a grounds-based laws to permitting 1^st^ trimester abortions on request is associated with a 7.8% (SD 3.7%) decrease in births due to unplanned pregnancies. This decline is driven by a fall in fertility among women 20-34 years old with secondary education. | Overall, the findings from 2 studies indicate that grounds-based laws may indirectly contribute to continuation of pregnancy and thus increased fertility. When grounds-based laws are removed, and 1^st^ trimester abortion is allowed on request, these studies demonstrated a decrease in fertility, possibly due to a reduction in unplanned births. |  |
| Clarke 2016 | ▲ | Shifting from a grounds-based laws to permitting 1^st^ trimester abortions on request and free of charge, is associated with reductions in fertility. Among women aged 15-44, fertility rates declined by 2.3 to 3.8% two years following the policy change. This trend was more marked among younger women aged 15-19 among whom fertility rates reduced between 5.1-7.1%. |  |  |
| **OUTCOME: OPPORTUNITY COSTS** | | | |  |
| Aiken 2018^1^ | ▲ | Grounds-based laws (real and substantial risk to life only) may contribute to opportunity costs for women who choose to travel to access an abortion, as well as those who choose self-managed abortion. Out of 38 women interviewed, 27 had chosen self-managed abortion and 11 had chosen to travel abroad. Some women who travelled reported experiencing emotional stress, trauma and financial costs. Women who travelled and those who chose self-managed abortion feared seeking post abortion care in their own country when experiencing complications due to anticipated judgement; those who self-managed also feared being reported to the authorities. | Overall, the findings from 15 studies, suggest that grounds-based laws may contribute to opportunity costs in several ways including: the need to travel for an abortion, increased financial costs, emotional stress and trauma, fear of/experienced judgement and stigma, bureaucratic and costly protracted legal processes, increased morbidity, being subjected to “interrogations” and having one’s rape claim questioned, unsafe abortions, having to carry an unwanted pregnancy or a pregnancy with severe malformations, to term. The findings from some of these studies point to an inconsistency in how grounds are interpreted and applied, which sometimes leads to unpredictability and inequity in terms of abortion access and healthcare quality for the abortion seeker. The findings from other studies indicate that certain grounds, such as health and rape grounds, are consistently interpreted very restrictively, which ultimately leads to the denial of an abortion. |  |
| Aiken 2019^1^ | ▲ | Grounds-based laws (preserve life and prevent grave and permanent damage to physical or mental health) may force women who do not meet one of the few legal exceptions, to travel abroad for an abortion or to self-manage their abortions. Out of 30 women interviewed, 11 travelled,18 self-managed and one qualified for a legal abortion. Women who travelled for an abortion reported emotional stress and financial costs. Women who opted for self-managed abortion reported fear of legal repercussions, confiscation of medications by customs and judgement, and emotional stress. |  |  |
| Amado 2010^2^ | ▲ | Under grounds-based laws (life, rape, incest, health, fatal foetal malformations, “unwanted insemination”), a review of 46 cases show that abortion seekers are sometimes verbally abused, and/or denied services. In 36 cases, the woman was denied an abortion or obtained an abortion only after a protracted bureaucratic process, of those 50% (n=18) sought abortion on rape grounds. Reasons for denial and/or delays were unjustified and included requests for legal or other authorization not required by law, institutional conscientious objection, ignoring court rulings, ignorance of the law, misinterpretation of the life and health ground, and questioning of women’s rape claims. |  |  |
| Arnott 2017^2,4^ | ▲ | Where grounds-based laws apply (life, health, rape, incest, age below 15/unable to consent to sex), abortion services may still be unavailable. Senior medical administrators who do not support abortion may limit access to abortion services by controlling the purchase of equipment and commodities, and by narrowly interpreting the mental health exception. Availability of services may thereby vary considerably between institutions and regions.^3^ |  |  |
| Black 2015^1^ | ▲ | In settings where grounds-based laws apply (threat to life*) and where local ethics committees are assigned the responsibility to determine legal eligibility for pregnancies >20 weeks or “in complex cases”, the process women go through in which their case is examined is sometimes lengthy, financially burdensome, and contributes to abortion delays.  *Defined through case law as including threats to physical and mental health, including changes to future social and/or economic circumstances that might affect health. |  |  |
| De Piñeres 2017^1^ | ○ | Where grounds-based laws apply (rape, incest, fatal foetal anomaly, life, health), women report significant challenges in obtaining care when they cannot obtain legal support and counselling. In a study among 21 women who were first denied a legal abortion, 5 out of 8 women who received legal support and counselling reported that they accessed an abortion; they felt that the support was crucial as it allowed them to advocate for themselves and navigate a complex system. |  |  |
| Diniz 2014^5^ | ▲ | Where grounds-based laws apply (rape, life), women who are legally eligible for an abortion may not be able to access one. Even when the law provides that a woman's claim of rape is sufficient to satisfy requirements for legal ground, only 13.7% (n=232) of participants (physicians) reported that they required only the woman’s narrative to grant her an abortion; almost half (44.1%, n=754) required at least one document not required by law such as a police report, judicial authorisation, expert external medical opinion or authorisation of institutional ethics committee and 37% (n=625) required a woman to present two or more such documents.  Interviews with physicians revealed that some providers frequently tested women’s rape claims by subjecting them to several "interviews", requiring them to have multiple ultrasounds to verify the chronology of the rape claim, by requiring women to provide legal documentation, or by requiring that they exhibit signs of psychological trauma in order to verify the "truth" for her to be granted a legal abortion. |  |  |
| Küng 2018^4^ | ▲ | In settings where abortion is permitted on health grounds, modes of interpretation and wording of health grounds can result in wide variations in availability under such a ground. This can contribute to opportunity costs through abortion delay or denial.  Some participants (providers and NGO workers) reported that very restrictive interpretation of the health ground meant that few abortions on this ground were granted. Incorrect interpretation and application of the law by healthcare providers may be due to limited knowledge about the law, stigma or personal negative attitudes towards abortion among physicians who´s responsibility it is to determine the risk to health. |  |  |
| LaRoche 2021^1^ | ▲ | Across a variety of contexts with grounds-based laws, some in combination with criminalisation, abortion seekers report that they are asked to justify their abortion and to fit it into one of the prescribed legal grounds. Some women experienced this as stigmatising and felt this created a hierarchy of deservingness and imbued a sense of judgment about their decision. |  |  |
| Madeiro 2016^2,6^ | ▲ | Where a grounds-based laws apply (rape, life) abortion accessed on a rape ground may still be very difficult to access. These delays or denials represent important opportunity costs of time and financial costs.  Even though not required by law, out of 37 institutions that reportedly provided abortions on rape grounds, 92% (n=34) reported that they required additional documentation to grant the abortion; 14% (n=5) required a police report, 8% (n=3) required a forensic report, 8% (n=3) required a court order, 11% (n=4) required an opinion from an institutional review board, and 8% (n=3) required an order from the department of public prosecutions. |  |  |
| Maira 2019^4^ | ▲ | Where grounds-based laws apply (life, rape, fatal foetal anomaly), access to an abortion may depend on the understanding of the law and its application by healthcare professionals. Participants reported that there are sometimes disagreements within a medical team about whether a foetal anomaly is incompatible with life (ground) or whether there is a risk to life (ground). In these cases, abortion is denied or delayed until the health condition is severe enough that health professionals can agree that the condition is life threatening.  In addition, healthcare providers report that in cases of rape, some providers impose additional requirements in order to provide the abortion. |  |  |
| McLean 2019^1^ | ▲ | Where grounds-based laws apply (rape, incest, life, health, severe foetal anomaly, “mentally unfit to bring up a child”), some healthcare providers perceive it as their task to determine the legitimacy of the abortion. Abortion seekers who are perceived to be lying about being raped or to not have a “good enough” reason to have an abortion even when they are legally eligible, may be denied one. |  |  |
| Påfs 2019^1^ | ▲ | Where grounds-based laws apply (life, rape, health, life, forced marriage), the abortion seeker has to prove her rape claim. Healthcare providers reported that going through the legal process is so time consuming, laborious and costly that few choose to go down this route. None of the interviewed participants had ever heard of a woman obtaining an abortion on the rape ground. In addition, some participating providers had limited knowledge about the law, and several were uncertain about how the law should be interpreted and applied. |  |  |
| Ramos 2014^2,6^ | ▲ | Where grounds-based laws apply (life, health, rape, mental disabilities) some healthcare providers have poor awareness and understanding of the law; out of 157 healthcare providers, 56.3% were not aware of the mental health ground and 24.1% were unsure/did not respond; 64% did not have a correct understanding of the rape ground and 24.1 % were unsure/did not respond.  Over half of respondents said that providers do not perform abortions because of restrictive interpretation of the law, hospitals requiring judicial authorisations, and lack of familiarity with the indications. |  |  |
| Sahin Hodoglugil 2017^4^ | ▲ | Where grounds-based laws apply (life, rape, forced marriage, incest), the process of obtaining a court order needed to access a legal abortion on rape grounds is so challenging, costly and lengthy that few choose to go down this path and instead end up carrying their pregnancies to term or having unsafe abortions. The evidence burden lies with the person who has been raped who must prove the rape to the courts. Out of 11 reviewed cases where abortion was sought on the rape ground, only one presented a court order and obtained an abortion. |  |  |
| **OUTCOME: SELF-MANAGED ABORTION** | | | |  |
| Aiken 2018^1^ | ▲ | Where grounds-based laws apply (real and substantial risk to life only), some women, who do not satisfy one of the prescribed legal grounds, may prefer and choose to self-manage their abortion over traveling to obtain abortion (27 out of 38 women interviewed). | Overall, evidence from 2 studies suggest that grounds-based laws may contribute to self-managed abortion. |  |
| Aiken 2019^1^ | ▲ | Where grounds-based laws apply (preserve life and prevent permanent damage to physical or mental health), some women, who do not satisfy one of the prescribed legal grounds, may prefer and choose to self-manage their abortion over traveling (18 out of 30 women interviewed). Some choose self-management as they cannot afford to travel. |  |  |
| **OUTCOME: UNLAWFUL ABORTION** | | | |  |
| Aiken 2018^1^ | ▲ | Where grounds-based laws apply (real and substantial risk to life only) some women who do not satisfy one of the prescribed legal grounds, go on to have unlawful self-managed abortion. Out of 38 women interviewed, 27 had chosen self-managed abortion. | Overall, evidence from 3 studies suggest that grounds-based laws may contribute to unlawful abortion. |  |
| Aiken 2019^1^ | ▲ | Where grounds-based laws apply (preserve life and prevent grave and permanent damage to physical or mental health) some women who do not satisfy one of the prescribed legal grounds, may go on to have unlawful self-managed abortion. Out of 30 women interviewed 18 chose to self-manage their abortions unlawfully. |  |  |
| Payne 2013^1^ | ▲ | Where grounds-based laws apply (health, life, foetal anomaly, rape), some physicians feel that the ambiguous nature of the law may contribute to unsafe clandestine abortions.^3^ |  |  |
| **OUTCOME: REPRODUCTIVE COERCION** | | | |  |
| McLean 2019^1^ | ▲ | Where grounds-based laws apply (rape, incest, life, health, severe foetal anomaly, “mentally unfit to bring up a child”), some healthcare providers perceive it as their task to determine the legitimacy of the abortion. Abortion seekers who are perceived to be lying about being raped or to not have a “good enough” reason to have an abortion even when they are legally eligible, may be denied one. | Overall, the findings from 2 studies suggest that grounds-based laws may contribute to reproductive coercion through the denial of an abortion. |  |
| Mirlesse 2013^1^ | ▲ | Where grounds-based laws apply (rape and foetal anomaly) and the ground for abortion in cases of foetal malformations requires the condition to be fatal, women with pregnancies with severe but non-lethal foetal malformations, are not given any other choice than to continue the pregnancy. |  |  |
| **OUTCOME: DISPROPORTIONATE IMPACT** | | | |  |
| Arnott 2017^7^ | ▲ | Where grounds-based laws apply (life, health, rape, incest, age below 15/unable to consent to sex), there are sometimes large variations between regions and institutions with regards to how liberal or restrictive the law is interpreted. This may contribute to disproportionate impact for women seeking an abortion in a setting where the law is interpreted very narrowly and where there is limited use of the mental health ground. | Overall, the findings from 5 studies suggest that grounds and grounds-based laws may have a disproportionate, negative impact on women with fewer resources, rural women and women with lower education, as well as those seeking abortion due to rape and on health grounds. |  |
| Casas 2017^1^ | ▲ | Where abortion is not legal on any grounds, women with few resources are disproportionately impacted as they cannot afford to travel abroad for a legal abortion, afford a safe abortion within the private healthcare sector, or afford professional counselling when experiencing mental health issues related to carrying a pregnancy with severe foetal malformations. |  |  |
| Diniz 2014^2,5^ | ▲ | Where grounds-based laws apply (rape, life), a study among physicians revealed that women and girls who seek an abortion due to rape are disproportionately disadvantaged as they are more likely (than other grounds) to face difficulties in accessing a legal abortion, particularly as their rape claim is often questioned and services are denied and or delayed. Out of 1690 physicians, only 13.7% (n=232) reported that they only required the woman’s narrative in order to grant an abortion. |  |  |
| Küng 2018^4^ | ▲ | In settings where abortion is permitted on health grounds, modes of interpretation and wording of health grounds can result in wide variation in availability under such a ground, with a resultant disproportionate negative impact on rural and poor women who may not have access to accurate information and are unable to travel for an abortion. |  |  |
| Mirlesse 2013^1^ | ▲ | Where grounds-based laws apply (rape and foetal malformation) and authorisation from a court is required, abortion in cases of fatal foetal malformation may be especially difficult to access for women who are poor or have lower education levels. |  |  |

▲ = the intervention leads to an increase in the outcome; ○ = the intervention leads to no change in the outcome; ∇ = the intervention leads to a decrease in the outcome. Symbol does not indicate magnitude or certainty of effect

^1^ Qualitative study design: tests of statistical significance not applicable

^2^ The study was not powered to look at this outcome.

3 Concerns about adequacy exist – data underlying the finding is not sufficiently rich, data come from a small number of studies and few participants

4 Mixed methods study design; qualitative component

5 Mixed methods study design; data from qualitative and quantitative component

6 Mixed methods study design; data from quantitative component

^7^ Mixed methods study design: unclear if data is based on qualitative of quantitative component

*Supple Table 2. Evidence Table:* The impact of the intervention on health professionals

| **OUTCOME: WORKLOAD IMPLICATIONS** | | | |
| --- | --- | --- | --- |
| **Studies** | **Direction of the evidence** | **What does this mean?** | **Overall conclusion** |
| Casas 2017^1^ | ▲ | Where abortion is not legal under any grounds, interviews with healthcare providers revealed that some providers express frustration with the fact they can diagnose severe fatal and non-fatal foetal malformations but they cannot help the pregnant woman obtain an abortion when she wants one. | Overall, the findings from 5 studies suggest that grounds and grounds-based laws may have workload implications including: difficulties in interpreting and applying the law, preparing detailed files for court reviews, stress and fear of legal repercussions, and a frustration with the system when a diagnosis of a non-lethal foetal malformation can be made but abortion is not permitted. |
| McLean 2019^1^ | ▲ | Where grounds-based laws apply (rape, incest, life, health, severe foetal anomaly, “mentally unfit to bring up a child”), some healthcare providers struggle to interpret and apply the law, especially in cases where the women is legally eligible but the provider does not perceive that the reason is “good enough”. |  |
| Mirlesse 2013^1^ | ▲ | Where abortion is legal only in cases of rape and foetal malformation, and authoriSation from a court is required to obtain an abortion, this may have workload implications as the medical referral centre has to prepare a detailed file for the court to review.  Where a ground exists for only lethal malformations, severe non-lethal foetal malformations are particularly challenging for physicians; some consider that their "hands and feet are tied" as they are not legally allowed to help the women with an abortion. |  |
| Payne 2013^1^ | ▲ | Where grounds-based laws apply (health, life, foetal anomaly, rape), some physicians, whose authority it is to determine satisfaction of prescribed grounds, interpret the health ground ambiguously. Ambiguity in how the law should be interpreted caused stress among some physicians who feared legal repercussions in case their judgement was challenged. Other physicians perceived the ambiguity as something positive as it increased access to legal abortion. |  |
| Ramos 2014^2^ | ▲ | Where grounds-based laws apply (life, health, rape, mental disabilities) some providers struggle with understanding and applying the mental health ground more than other grounds. |  |
| **OUTCOME: REFERRAL TO ANOTHER PROVIDER** | | | |
| Black 2015^1^ | ▲ | Where grounds-based laws apply (threat to life, but possibly can be interpreted based on case law to include threats to physical and mental health, including changes to social and economic circumstances that might affect health) and where local ethics committees are assigned the responsibility to determine legal eligibility for women with pregnancies >20 weeks or “in complex cases”, the process women go through in which their case is examined is sometimes protracted. 20 out of 22 providers reported that they or their colleagues had referred women to travel to another state to other providers because of how long the process is to summon the ethics committee and obtain a legal abortion in the present state. | Overall findings from 1 study suggest that grounds-based laws may contribute to referrals to another provider; physicians must make referrals to providers in another state to circumvent existing obstacles including ethics committees and other protracted processes. |
| **OUTCOME: IMPOSITION ON CONSCIENCE OR ETHICS** | | | |
| Casas 2017^1^ | ▲ | Where abortion is not legal under any ground, interviews with healthcare providers reveal that some providers express frustration with the fact they can diagnose severe fatal and non-fatal foetal malformations but they cannot help the pregnant woman obtain an abortion when she wants one. | Overall, the findings from 2 studies indicate that grounds and grounds-based laws may contribute to providers experiencing an imposition on their conscience or ethics in two ways, either by a) resulting in the questioning of whether or not a provider should provide a legal abortion, or b) by preventing providers from giving women diagnosed with a foetal malformations an option to end their pregnancy. |
| McLean 2019^1^ | ▲ | Where grounds-based laws apply (rape, incest, life, health, severe foetal anomaly, “mentally unfit to bring up a child”), some healthcare providers struggle to interpret and apply the law, especially in cases where the women is legally eligible but the provider does not perceive that the reason is “good enough” or when the patient is thought to be lying about their rape claim. Providers felt that these cases forced them into ethically challenging situations whether to provide the abortion or not. |  |
|  |  |  |  |
| **OUTCOME: STIGMATISATION** | | | |
| Madeiro 2016^1^ | ○ | Where grounds-based laws apply (preserve life, rape), providers may still be unwilling to provide abortion care to legally eligible women due to stigmatisation of abortion providers.^3^ | Overall, the findings from 1 study indicate that grounds-based laws may contribute to stigmatisation of healthcare providers who ultimately choose not to involve themselves in abortion care for this reason. |
| **OUTCOME: SYSTEM COST** | | | |
| Aitken 2017^3^ | ▲ | Grounds-based laws (real and substantial risk to life only) may contribute to increased system costs; 33 physicians reported having been involved in the management of a women who had an abortion because of severe or life-threatening illness. Of those, 27% reported that they had delayed the abortion until a pregnancy was deemed a "real and substantial risk" to life. | Overall, the findings from 5 studies suggest that grounds and grounds-based laws may contribute to system costs by indirectly contributing to continuation of pregnancy and maternal mortality, and directly by imposing costs on court systems, increased workloads of healthcare professionals, and by delaying care for pregnant women with severe health conditions. |
| Antón 2018^3^ | ▲ | Shifting from a grounds-based laws to permitting 1^st^ trimester abortions on request is associated with an 7.8% (SD 3.7%) decrease in births due to unplanned pregnancies. This decline is driven by a fall in fertility among women 20-34 years old with secondary education. |  |
| Casas 2017^1^ | ▲ | Where abortion is not legal under any ground, women are forced to carry non-viable and medically high-risk pregnancies to term. This may contribute to system costs by increasing the healthcare resources needed to provide obstetrical care and increasing the risk of maternal morbidity and mortality. |  |
| Clarke 2016 | ▲ | Shifting from a grounds-based laws to permitting 1^st^ trimester abortions on request and free of charge, is associated with reductions in maternal mortality. Following the policy change maternal mortality fell by 8.8-16.2% among women aged 15-44 and by 14.9-30% among adolescents. |  |
| Mirlesse 2013^1^ | ▲ | Grounds-based laws (rape) may contribute to system costs where authorisation from a court is required in cases of lethal foetal malformations. A detailed file must be prepared by the medical referral centre which requires significant work. |  |

▲ = the intervention leads to an increase in the outcome; ○ = the intervention leads to no change in the outcome; ∇ = the intervention leads to a decrease in the outcome. Symbol does not indicate magnitude or certainty of effect

^1^Qualitative study design: tests of statistical significance not applicable

^2^ Mixed methods design; from qualitative component

^3^ Study is not powered to look at this outcome and indirectly assesses systems costs. The study uses as a proxy delay in abortion until maternal condition is significantly affected.

**Appendix: Search Strategy**

**Limitations**

- Year: from 2010
- Study designs: no restrictions, PhD thesis ok
- Language or geographics: No restrictions

**Databases:** Pubmed, HeinOnline, JStore, Google Scholar

**Concepts:**

1. Abortion
2. Laws//regulations/policies

Pubmed

|  | **#** | **Searches** | **Results** |
| --- | --- | --- | --- |
| **Abortion** | 1 | "abortion, induced"[MeSH Terms] OR "abort*"[Title/Abstract] OR "abortion, criminal"[MeSH Terms] OR “termination of pregnancy”[Title/Abstract] OR “menstrual regulation” [Title/Abstract] OR “pregnancy termination”[Title/Abstract] | 99131 |
| **Laws//regulations/policies** | 2 | “Law enforcement”[mesh] OR “Criminal Law”[mesh] OR “policy” [mesh] OR “policy” [Title/Abstract] OR “policies”[Title/Abstract] OR “regulation*”[Title/Abstract] OR “legal ground*”[Title/Abstract] OR “grounds-based”[Title/Abstract] OR “health threat”[Title/Abstract] OR “life threat”[Title/Abstract] OR “fetal anomaly”[Title/Abstract] OR “fetal condition”[Title/Abstract] OR “congenital abnormalit*”[Title/Abstract] OR “foetal”[Title/Abstract] OR “rape”[Title/Abstract] OR “incest”[Title/Abstract] OR “disability*”[Title/Abstract] OR “cognitive impairment”[Title/Abstract] OR “intellectual impairment” [Title/Abstract] OR “economic ground*”[Title/Abstract] OR “social ground*”[Title/Abstract] OR “socio-economic ground*”[Title/Abstract] OR “on request”[Title/Abstract] OR “save life” [Title/Abstract] OR “preserve health”[Title/Abstract] | 1,508395 |
| **1+2**  **From 2010** |  |  | 9785  3183 |

**JSTOR**

"abort*" OR “termination of pregnancy” OR “pregnancy termination” AND “Law?” OR “Policy” OR “policies” OR “regulation?” OR “legal ground?” OR “grounds-based”

**125 citations. After limiting from 2010: 45**

**HeinOnline**

"abort*" OR “termination of pregnancy” OR “pregnancy termination” OR “menstrual regulation” AND “Law?” OR “Policy” OR “policies” OR “regulation?” OR “legal ground?” OR “grounds-based” OR “health threat” OR “life threat” OR “fetal anomaly” OR “fetal condition” OR “congenital abnormalit*” OR ab:“foetal” OR ti:”foetal” OR “rape” OR “incest” OR “disability*”OR “cognitive impairment” OR “intellectual impairment” OR “economic ground*”OR “social ground*”OR “socio-economic ground*”OR “on request” OR “save life” OR “preserve health”

**Google Scholar**

"abortion" OR “termination of pregnancy” OR “pregnancy termination” OR “menstrual regulation” AND “Law” OR “Policy” OR “policies” OR “regulation” OR “legal ground” OR “grounds-based” OR “health threat” OR “life threat” OR “fetal anomaly” OR “fetal condition” OR “congenital abnormality” OR “foetal” OR “rape” OR “incest” OR “disability*”OR “cognitive impairment” OR “intellectual impairment” OR “economic ground” OR “social ground” OR “socio-economic ground” OR “on request” OR “save life” OR “preserve health”

**7530. After limiting from 2010: 3360**

**Imported from publish and perish – 997 + 995**
